# From Zn(II) to Cu(II) Detection by MRI Using Metal-Based Probes: Current Progress and Challenges

**DOI:** 10.3390/ph13120436

**Published:** 2020-11-30

**Authors:** Kyangwi P. Malikidogo, Harlei Martin, Célia S. Bonnet

**Affiliations:** Centre de Biophysique Moléculaire, Université d’Orléans, Rue Charles Sadron, F-45071 Orléans 2, France; pmalikidogo@gmail.com (K.P.M.); harlei.martin@cnrs-orleans.fr (H.M.)

**Keywords:** zinc detection, copper detection, MRI, responsive contrast agents, lanthanides, molecular imaging, quantification, paraCEST, parashift, relaxation

## Abstract

Zinc and copper are essential cations involved in numerous biological processes, and variations in their concentrations can cause diseases such as neurodegenerative diseases, diabetes and cancers. Hence, detection and quantification of these cations are of utmost importance for the early diagnosis of disease. Magnetic resonance imaging (MRI) responsive contrast agents (mainly Lanthanide(+III) complexes), relying on a change in the state of the MRI active part upon interaction with the cation of interest, e.g., switch ON/OFF or vice versa, have been successfully utilized to detect Zn^2+^ and are now being developed to detect Cu^2+^. These paramagnetic probes mainly exploit the relaxation-based properties (T_1_-based contrast agents), but also the paramagnetic induced hyperfine shift properties (paraCEST and parashift probes) of the contrast agents. The challenges encountered going from Zn^2+^ to Cu^2+^ detection will be stressed and discussed herein, mainly involving the selectivity of the probes for the cation to detect and their responsivity at physiologically relevant concentrations. Depending on the response mechanism, the use of fast-field cycling MRI seems promising to increase the detection field while keeping a good response. In vivo applications of cation responsive MRI probes are only in their infancy and the recent developments will be described, along with the associated quantification problems. In the case of relaxation agents, the presence of another method of local quantification, e.g., synchrotron X-Ray fluorescence, single-photon emission computed tomography (SPECT) or positron emission tomography (PET) techniques, or ^19^F MRI is required, each of which has its own advantages and disadvantages.

## 1. Introduction

Zinc and copper are essential cations involved in many fundamental biological processes and their concentration is highly regulated in living systems. Any disturbance in their homeostasis is involved in pathologies such as neurodegenerative diseases, diabetes, cancer and Wilson and Menkes diseases [1,2,3,4,5,6].

Zinc is the second most abundant transition metal in the body after iron and most biological zinc is involved in structural and catalytic elements, which are essential for the structure of metalloproteins and play an important role not only in regulation and/or activation, but also in gene transcription. It is the only element that is present in all classes of enzymes. It is also found in large quantities in the brain (up to 300 µM in the vesicles of some neuronal glutamatergic cells) [7], where it plays a crucial role in the transmission of information. In addition, Zn^2+^ interacts with many types of ion channels [8,9]. From a neuropathological point of view, although ionic zinc is considered as an endogenous modulator of synaptic activity and neuronal function, uncontrolled exposure to a large amount of labile Zn^2+^ can lead to excitotoxic neuronal death. This type of cell death occurs during epileptic seizures, head trauma, cerebral ischemia and perfusion, but also in situations related to very intense neuronal activity [10,11,12].

Zn^2+^ is implicated in the formation of amyloid plaques such as β-amyloid involved in Alzheimer’s disease [13], but also islet amyloid polypeptide linked to type 2 diabetes [14]. In fact, Zn^2+^ is co-released with insulin from pancreatic β-cells in response to glucose stimulation. It has been shown that inhibition of the islet amyloid polypeptide aggregation by insulin depends on the insulin oligomeric state, which is in turn regulated by zinc concentration [15]. Zn^2+^ is also implicated in various cancers, such as breast, pancreas or prostate [16]. The prostate contains the highest concentration of zinc of all soft tissues and secretes high amounts of Zn^2+^ into the prostatic fluid. Zinc levels in malignant prostate are markedly reduced, suggesting that high concentrations of zinc may be essential for maintaining a healthy prostate. Furthermore, evidence now shows that zinc may have an antitumor activity role in prostate cancer. Therefore the detection of zinc levels in prostate tissues is urgently needed for the early detection of prostate cancer and a better chance of survival [17].

Copper is the third most abundant transition metal in the body, after zinc and iron. It is redox-active with the presence of Cu^+^ (mostly intracellular) and Cu^2+^ (predominantly in the extracellular media) in biological systems. Therefore, it serves as a redox-active cofactor in many enzymatic processes in eukaryotic cells, including for example cytochrome c oxidase (for electron transfer), or superoxide dismutase (for redox catalysis). It is also involved in other proteins such as metallothioneins (for metal ions detoxification) or ceruloplasmin (for Cu delivery and excretion), and it is an activating factor for many enzymes such as catalase, or glutathione peroxidase. However, due to its redox activity, Cu is also prone to generate reactive oxygen species, which causes catastrophic damage to lipids, proteins and DNA [18]. Consequently, Cu is tightly regulated in cells and in multicellular organisms.

The inadequate regulation of Cu homeostasis and/or the poor distribution to cells and tissues [19] can lead to a number of serious diseases [20]. For example, Wilson and Menkes diseases are caused by Cu accumulation in the liver and intestines, respectively, resulting in a systemic deficiency. Cu is also responsible for the formation of β-amyloid plaques in Alzheimer’s disease and is implicated in prion diseases such as Creutzfeld–Jacob’s. Furthermore, Cu is associated with cancers due to the generation of free radicals, which can play a role in the proliferation of malignant cells. It has been demonstrated that cancer cells need higher Cu concentration to grow compared to normal cells, with high serum and tissue Cu concentrations found in various types of cancers such as brain, breast and liver cancers and leukemia [21].

Consequently, the detection and quantification of Zn^2+^ and Cu^2+^ in the extracellular media are of prime importance for the early diagnosis of these Cu- and Zn-related diseases. Although classical analytical techniques are available for the quantification of these ions, imaging techniques showing the presence/location of the labile pool in vivo in real-time would be a huge advantage for these early diagnoses. Accordingly, there has been tremendous development in molecular imaging in the past ten years. Molecular imaging seeks to visualize the expression and function of bioactive molecules, highlighting the physiological abnormalities underlying the diseases, rather than their structural consequences, which often happen at a later stage. The use of an imaging probe is essential in molecular imaging and is the component that will be selectively influenced by the biomarker being detected. Extensive research has been carried out on the chemistry of these imaging probes for the detection of a wide range of biomarkers (pH, enzymatic activity, pO2, physiological cations and anions, temperature, etc.) [22,23,24].

Concerning metal ions and more precisely Cu^2+^ and Zn^2+^, the main imaging techniques so far relies on optical detection [25,26,27]. Although very sensitive and adapted to surface imaging or guided-surgery, it is not possible to image every part of the body with optical imaging as light does not penetrate sufficiently. Moreover, this technique suffers from a lack of macroscopic resolution. On the contrary, Magnetic resonance imaging (MRI) is characterized by a high spatial and temporal resolution, with no depth penetration limitations making it possible to image the whole body. It suffers however from a lack of sensitivity and the current limit of detection of MRI contrast agents in clinics is ca. 0.1 mM. Therefore, it is of prime importance to know the average concentration of the targeted metal ion to select the most appropriate/promising imaging technique.

In the extracellular media, both Cu^2+^ and Zn^2+^ are found as a static pool, for which M^2+^ is closely linked to various metalloproteins, and a mobile or exchangeable pool of free or loosely bound/kinetically labile M^2+^. Cu extracellular concentrations are 10–25 µM in serum, 0.5–2.5 µM in cerebrospinal fluid and 30 µM in the synaptic cleft [28]. Zn^2+^ concentrations are generally higher, reaching 10 mM in prostatic fluid, 10–20 mM in β-cells of the pancreas, 300 µM in vesicles of glutamatergic neuronal cells and around 10 µM in blood serum [29]. These relatively high concentrations are therefore adapted to the relatively low sensitivity of MRI, although in the case of Cu^2+^, we are at the lowest limit of detection.

## 2. Metal-Based Contrast Agents in MRI

Paramagnetic metal ions are particularly interesting as they can be used in MRI either for their relaxation properties or their paramagnetic-induced shift properties, or both. In this perspective, the chemistry of lanthanide ions is particularly exciting. Lanthanides correspond to the first period of the f-block elements and form stable Ln^3+^ cations possessing characteristic 4f^n^ open-shell configuration. Because these f-electrons are quite shielded by the 5s^2^ and 5p^6^ electrons, they are not very affected by external perturbations or involved in covalent interactions, leading to versatile coordination behavior. They have similar chemical behaviors along the series, but possess unique physical, spectroscopic and magnetic properties.

For example, Gd^3+^ due to its high electronic spin (7/2) and relatively long electronic relaxation is a choice ion for developing T_1_-contrast agents. These agents affect the longitudinal relaxation time of water protons in surrounding tissues and give a positive contrast on MRI images. The lanthanide paramagnetic-induced hyperfine shifts (with the exception of Gd^3+^) have also been used for paramagnetic chemical exchange saturation transfer (ParaCEST) or paramagnetically shifted (Parashift) probes. They also have unique optical properties and several isotopes that can be detected by nuclear imaging techniques. In short, the choice of the Ln^3+^ will dictate the imaging technique to use and while similar coordinating ligands can be foreseen, small modifications on the ligands will be needed to meet the requirements of a given technique.

Although some examples of cation detection have been achieved using hyperpolarized ^129^Xe [30] or diaCEST (diamagnetic CEST, in particular, ^19^F CEST) [31,32], the vast majority of Cu^2+^ and Zn^2+^ MRI responsive contrast agents are based on paramagnetic complexes (mainly lanthanide(+III) complexes but also transition metal complexes), on which we will focus this review.

**Figure 1:**
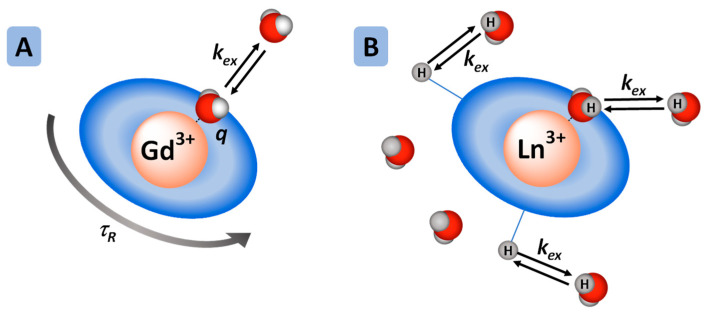
(**A**) Principal microscopic parameters that influence the relaxivity of Gd^3+^-based contrast agents. (**B**) ParaCEST Ln^3+^ complex: the ParaCEST effect originates from proton exchange between the bulk water and protons on the ligand or on the coordinated water molecules.

The theory of Gd^3+^-based MRI contrast agents has been extensively described elsewhere [33]. The efficacy of contrast agents is called relaxivity (paramagnetic relaxation enhancement of water proton per millimolar concentration of Gd^3+^). Due to their toxicity, Gd^3+^ ions must be used as thermodynamically stable and kinetically inert complexes. Their efficacy is related to the microscopic parameters of the Gd^3+^ complexes (Figure 1A). The most important microscopic parameters (which the chemist can alter by careful design of the contrast agent) are:

(1) The number of water molecules directly coordinated to Gd^3+^, hydration number: *q*, must be at least one but not too high so that the thermodynamic stability remains sufficient;
(2) The exchange rate of these water molecules with the bulk: *k*_ex_;
(3) The rotational correlation time of the complex: τ_R_, linked to the size and rigidity of the system.

CEST agents possess exchangeable protons such as –OH of alcohols/water and –NH of amines/amides/carbamates, which are in slow exchange with those of the bulk (Figure 1B). The magnetization of the exchangeable protons is modified by a pre-saturation pulse, and due to the chemical exchange, the intensity of the water peak is decreased. The intensity of the water peak is then translated into an image. The CEST signal is therefore characterized by the chemical shift of the exchangeable protons, and their exchange rate with the bulk, *k*_ex_. In order to observe a CEST effect, *k*_ex_ must be lower than the chemical shift difference between the exchangeable proton and the water proton of the bulk. To explore faster exchange rates, the use of paramagnetic agents is highly interesting as the chemical shifts of the exchangeable protons will be shifted. This also limits the detrimental effect of relaxation, and to shift the CEST effect beyond the tissue magnetization transfer window. Several paramagnetic metals have been used to design ParaCEST agents, and more specifically responsive ParaCEST agents. For example, Fe^2+^ [34] and Co^2+^ [35,36] complexes have been used for pH detection, Co^2+/3+^ [37] systems have been designed as redox-active probes, but for cation detection, the ParaCEST probes developed are composed of paramagnetic Ln^3+^ (with the exception of Gd^3+^).

The paramagnetic-induced hyperfine shifts have also been exploited in the field of parashift probes. These are paramagnetically shifted MRI probes, which have proton nuclei (not exchangeable) near a paramagnetic center with resonances at frequencies shifted away from the usual diamagnetic range. It allows their visualization without any background signal. These protons are generally methyl or *tert*-butyl groups, but they can also be CH_2_ groups [38]. Ideally, the paramagnetically shifted proton resonances must be as far away as possible from the diamagnetic window, so that a large excitation bandwidth can be utilized to use fast imaging sequences. Moreover, the proximity of the paramagnetic center shortens the relaxation time of the proton, which can be used to enhance sensitivity through the use of fast-pulsed NMR techniques. Transition metal complexes of Fe^2+^ and Co^2+^ have been used as parashift agents especially for temperature sensing and more recently anion sensing, however, no imaging studies have been reported yet. Lanthanide complexes (Dy^3+^ and Tm^3+^) were successfully used to map pH and temperature in vivo.

## 3. Principle of Cation Detection

### 3.1. General Design and Requirements

**Figure 2:**
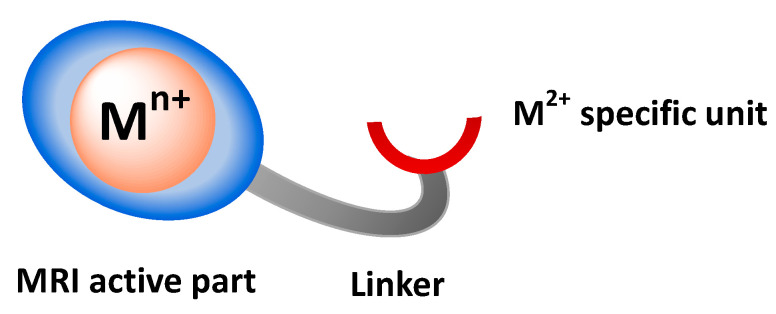
Design of cation responsive contrast agents.

The development of MRI contrast agents for molecular imaging will require a change in the state of the contrast agent upon interaction with the biomarker of interest: switch OFF/ON or vice versa. Rather than looking for maximum efficacy, a maximum change upon biomarker interaction will be needed to increase the sensitivity of detection. It is also easier to observe a switch ON signal rather than a switch OFF. The Zn^2+^ or Cu^2+^ responsive probes are typically composed of three main parts (Figure 2): a paramagnetic metal–ion complexing unit (MRI active part), an M^2+^ complexing unit (specific to the cation being detected), with a linker between them.

Most of the probes discussed below are Ln^3+^ complexes, and in this case polyaminopolycarboxylate ligands are preferred. Derivatives of macrocyclic DOTA (1,4,7,10-tetraazacyclododecane-1,4,7,10-tetraacetic acid) or linear DTPA (1,1,4,7,7-pentakis-(carboxymethyl)-1,4,7-triazaheptane) are widely, although not exclusively, used. They are chosen because of their high thermodynamic stability and kinetic inertness.

In addition to the previous requirements, the contrast agent must be (1) selective for the given cation, and even for a specific oxidation state in the case of Cu; (2) responsive to the cation in the physiologically relevant concentration range; (3) able to bind reversibly to the cation to prevent disruption of homeostasis. Finally, (4) M^2+^ complexation should affect the MRI active part in order to obtain a responsive probe. This last point is not easy to predict and will depend on the technique used (T_1_, paraCEST or shift MRI), and will be discussed separately in the following sections.

### 3.2. T_1_-Based Contrast Agents

**Figure 3:**
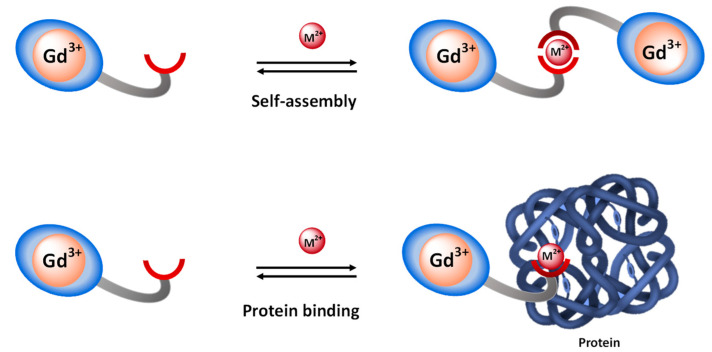
Principle of cation detection by τ_R_-modulation, achieved by either self-assembly or by protein binding.

The principle of detection by MRI is based on the modification of microscopic parameters influencing the efficiency of a contrast agent upon cation interaction. Since the chemist is able to predict a τ_R_ or *q* modification by careful design of the contrast agent, cation detection by MRI is mainly based on the modification of these two parameters. For detection through τ_R_ modulation, probes often undergo an increase in molecular weight and/or rigidity upon cation interaction, which results in an increase in τ_R_ and hence an increase in relaxivity, r_1_. Such increases in molecular weight usually occur due to self-assembly induced by the cation (M^2+^) or protein binding (Figure 3). The effect of τ_R_ is greater at intermediate fields (20–60 MHz), making the response more efficient in this field-range.

**Figure 4:**
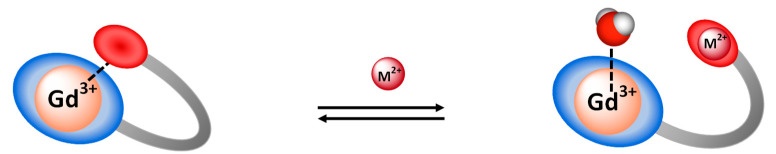
Principle of cation detection by *q*-modulation.

The design of contrast agents based on *q*-modulation focuses on the re-arrangement of the Gd^3+^ coordination environment upon cation interaction. A coordinating function should switch from Gd^3+^ to the cation (M^2+^), allowing for one (or more) water molecules to enter the first Gd^3+^ coordination sphere (Figure 4). This results in an increase in relaxivity. This “flipping mechanism” is not easy to predict as it will depend on the cation (M^2+^), the Gd^3+^ coordinating unit, the M^2+^ coordinating unit, and on the linker. The challenge is also to design a stable Gd^3+^ probe with or without the presence of the cation (M^2+^). A modification of *q* can also be expected when the cation detection occurs next to the Gd^3+^ coordination sphere and hinders the presence of the water molecule due to steric constraints. This results in decreasing relaxivity, causing the system to switch OFF.

### 3.3. ParaCEST and Parashift Contrast Agents

The development of responsive ParaCEST contrast agents relies on the modification of the CEST effect through cation interaction by a modification of the chemical shift of the exchangeable protons and/or their exchange rate with the bulk. The exchangeable protons can be present on the Ln^3+^ complexing unit, on the M^2+^ coordinating moiety, and/or on the linker (Figure 2). The rational design of such probes is not easy, as the efficacy of these agents will depend on the geometry/distance of the exchangeable protons both with respect to the Ln^3+^ and to the cation (M^2+^), with the aim of inducing an observable CEST change upon cation complexation. Ideally, the CEST signal must be switched ON or modified upon cation interaction.

The same principle holds for parashift agents as the chemical shift of the paramagnetic protons must be modified upon cation interaction.

## 4. Design of T_1_-Based Probes for Zn^2+^ and Cu^2+^ Detection

### 4.1. Generalities on the Design

T_1_-based probes for Zn^2+^ and Cu^2+^ detection are Gd^3+^ complexes, which are mainly, but not exclusively GdDTPA or GdDOTA complexes. It should be noted that the first examples of Zn^2+^ responsive probes were based on GdDTPA, but more recent studies showing the higher kinetic inertness of macrocyclic Ln^3+^ such as GdDOTA, particularly compared to GdDTPA-bisamide systems [39], prompted the discovery of a library of ligands based on macrocyclic scaffolds. The stability of those probes towards transmetallation is obviously of prime importance, and can be assessed easily using relaxometric studies in the presence of Zn^2+^ and PO_4_^3−^ [40]. This is even more crucial when the response to the cation is based on a *q*-change as the coordination sphere of the Gd^3+^ complex is affected.

The main difference between Zn^2+^ and Cu^2+^ contrast agents will be made on the specific binding unit, which must be selective for the desired cation vs other physiological cations, and in the case of Cu^2+^ for one oxidation state (+II) vs (+I). Due to its lower concentration in vivo, the selectivity is even more crucial for Cu^2+^ as a small response to Zn^2+^ will be very detrimental to achieve accurate Cu^2+^ detection. Zn^2+^ detection is often achieved using either iminodiacetate functions, or more often a bis-(2-pyridinemethyl) amine (DPA) unit, which is more selective for Zn^2+^ vs Ca^2+^ and which has a good affinity for Zn^2+^ (*K*_d_ ~ 25 nM) [41]. Similar functions will be often used to detect Cu^2+^ highlighting the selectivity issues which will be reviewed in detail below.

### 4.2. Small Molecular Probes for Zn^2+^ Detection

The development of Zn^2+^-responsive contrast agents has been tremendous in the last years, pushed forward by the success obtained in the first in vivo applications (vide infra) [42].

**Figure 5:**
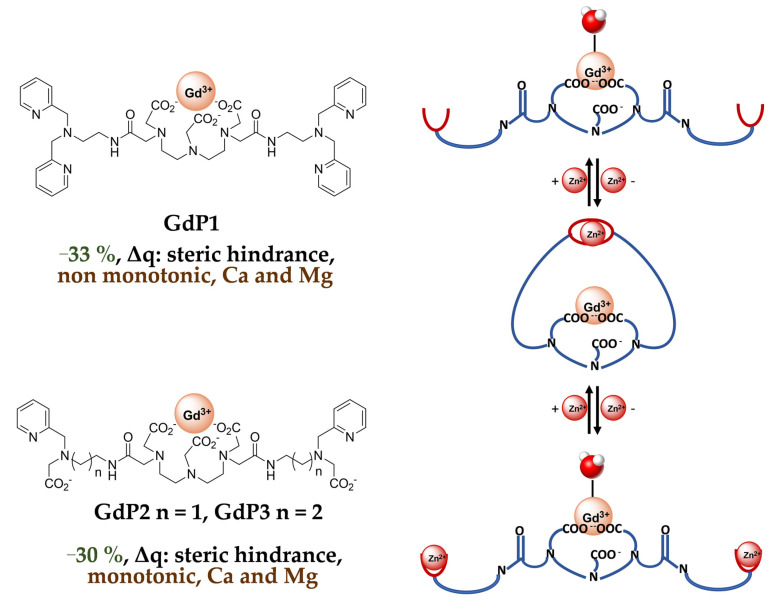
Zinc responsive contrast agents based on GdDTPA-bisamide systems developed by Nagano et al. In green (Δr_1_), black (mechanism of response), brown (response to Zn^2+^ (1 eq.) and selectivity).

The first examples of Zn^2+^-sensitive probes were developed by Nagano et al. and are based on GdDTPA-bisamide complexes, **GdP1, GdP2** and **GdP3 (**Figure 5). The Zn^2+^ complexing units are either two DPA units or modified DPA units, where one pyridine group has been replaced by a carboxylate function [43,44]. These contrast agents show a decrease in relaxivity when one equivalent of Zn^2+^ is added, transferring the Zn^2+^-binding to the top of the Gd^3+^ complex hindering the access of the water molecule to Gd^3+^ (Figure 5). However, in the case of **GdP1**, the addition of the second equivalent of Zn^2+^ leads to a further increase in relaxivity until it reaches its initial value. This can be explained by a modification of the complex geometry and coordination of each equivalent of Zn^2+^ to one DPA unit (formation of **GdP1Zn_2_**), providing space for the water molecule to access Gd^3+^ again. This explanation for the response of **GdP1** was hypothesized based on the behavior of a model compound bearing only one DPA unit, which does not show any relaxivity response to Zn^2+^. The non-monotonic response to Zn^2+^ of **GdP1** is problematic for in vivo use as one relaxivity corresponds to two Zn^2+^ concentrations. Therefore, in **GdP2** and **GdP3** the Zn^2+^-coordinating unit has been modified to reduce the formation of trinuclear complexes in the presence of two equivalents of Zn^2+^. This was clearly successful for **GdP2**, which shows a monotonic response to Zn^2+^, but **GdP3** does not respond to Zn^2+^, which highlights the importance of the length of the linker. Finally, it should be mentioned that the system is selective for Zn^2+^ vs Mg^2+^ and Ca^2+^, but the selectivity vs Cu^2+^ has not been reported.

The toxicity of the linear GdDTPA-bisamide systems **GdP1–3** has not been reported, but given the nature of the Gd^3+^ chelate (DOTA-bisamide), the transmetallation towards Zn^2+^ ions, as reported for similar systems [40], is expected to prevent practical applications in vivo.

**Figure 6:**
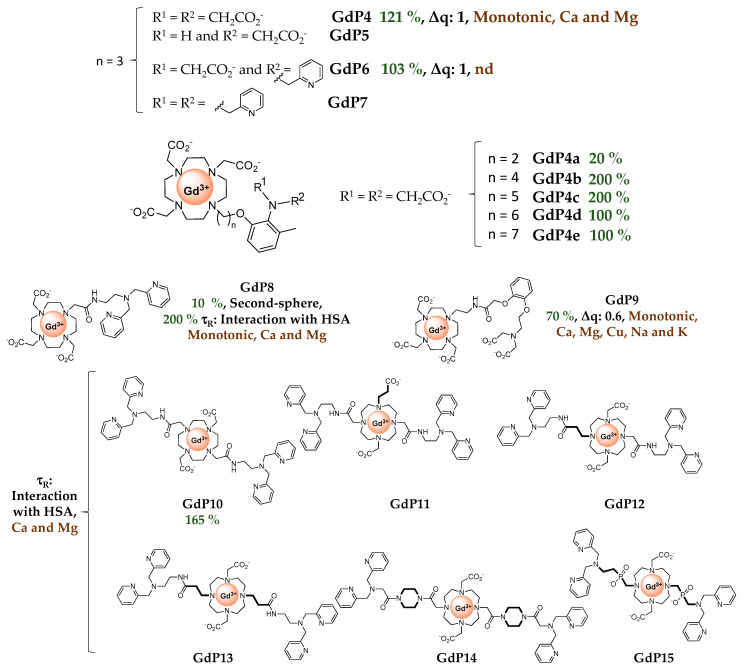

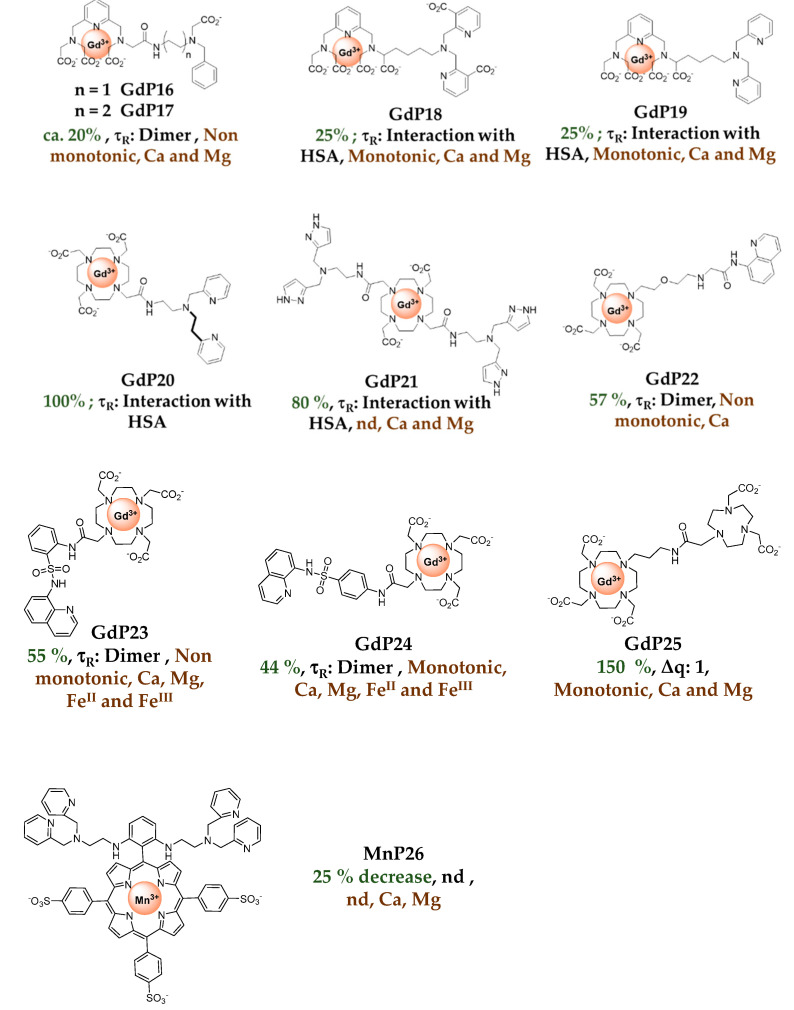
Metal-based complexes for Zn^2+^ detection: In green (maximum Δr_1_), black (mechanism of response), brown (type of Zn^2+^ response and selectivity).

Meade et al. have developed a series of GdDO3A-based contrast agents (DO3A = 1,4,7,10-tetraazacyclododecane-1,4,7-triacetic acid) with various Zn^2+^ complexing units (Figure 6 **GdP4–7**) [45,46,47]. **GdP4** shows a relaxivity increase of 121% upon Zn^2+^ binding, which was associated with a *q*-change as demonstrated by luminescence lifetime measurements on the corresponding Tb^3+^ complexes. In the absence of zinc, the carboxylate groups of Zn^2+^-binding moiety can weakly coordinate to Gd^3+^. Upon Zn^2+^ interaction, these carboxylate functions switch to Zn^2+^, allowing a water molecule to occupy the first coordination sphere of Gd^3+^. Systematic studies on similar complexes bearing different Zn^2+^-binding units **GdP5–7** have shown that two bindings groups including at least one carboxylate function must be present to generate a variation in the hydration number [46]. The same conclusion was observed for **GdP8** (Figure 6), where no *q*-change is observed upon Zn^2+^ binding, although it should be noted that one additional amide function is present in the coordination sphere of Gd^3+^ [48]. As in the aforementioned example with DTPA derivatives, the length of the linker is also an important parameter. The best responses are obtained with **GdP4b** and **GdP4c** where the alkyl chain contains four or five carbon atoms. Finally, for these systems containing an iminodiacetate moiety for Zn^2+^ binding, a good selectivity for Zn^2+^ vs Ca^2+^ and Mg^2+^ was observed. However, as a similar system was reported for Cu^2+^ detection, this structure does not provide good selectivity for Zn^2+^ vs Cu^2+^, which is often the case. A better selectivity was obtained for hexadentate **GdP9**, where the Zn^2+^-binding moiety contains a hexadentate group comprising an iminodiacetate moiety [49]. A *q*-increase is also observed in the presence of Zn^2+^, explaining the relaxivity increase. All these systems are based on DO3A chelates for Gd^3+^ complexation, and in the presence of Zn^2+^, the stability of the Gd^3+^ chelate, its kinetic inertness and the replacement of water molecules by physiological anions are important parameters to take into account for further in vivo studies.

Sherry et al. proposed to use two extended DPA motifs, namely BPEN in **GdP10** (Figure 6). Contrary to **GdP1**, the system did not prevent the access of the water molecule to Gd^3+^ in the presence of Zn^2+^. This observation could be explained by the local charge difference in the GdDTPA-bisamide complex (neutral) and GdDOTA-biasamide (+1). Interestingly, they have shown that this system is able to detect Zn^2+^ in the presence of human serum albumin (HSA). HSA is the most abundant protein in the blood serum (with a concentration of ca. 0.6 mM) and the cerebrospinal fluid [50]. It is composed of three domains (I, II, III), each one divided into two sub-domains (A and B) and it is used as a carrier for a variety of nutrients, metabolites and xenobiotics. It has two interacting domains for small molecules (such as Ibuprofen or warfarin), sites 1 and 2, which are located respectively in sub-domain IIA and IIIA. There are also fatty acid binding sites, as well as metal-binding sites. For example, the majority of the “free” or exchangeable pool of Zn^2+^ in plasma is bound to HSA. The high-affinity zinc-binding site is located at the interface of domain I and II, and the affinity has been determined to be in the µM range [51]. Hence, it is important to determine how contrast agents work in the presence of HSA, particularly for cation detection. **GdP10** is able to detect Zn^2+^ in the presence of HSA, relying on a different binding affinity of whether Zn^2+^ is present or not. Without Zn^2+^, the complex interacts weakly with HSA, whereas in the presence of this metal cation, the interaction is stronger. The mechanism of relaxivity modulation is based on a change in the overall rotational correlation time (τ_R_) of the complex as illustrated in Figure 3. It has been shown that the system is bound to HSA in site 2 of subdomain IIIA, and the affinity for Zn^2+^ (nM range), which is higher than the affinity of HSA for Zn^2+^, is consistent with the formation of **GdP10Zn** interacting with HSA. To improve the system, a series of complexes (**GdP11–15**) were developed to modulate the water exchange rate, which in the case of macromolecules is known to be the limiting factor. For all systems, the water exchange rate was improved, however, it does not always translate to a better Zn^2+^ response. The best systems are (**GdP11–12)**, which exhibit exceptionally high longitudinal relaxivities of approximately 50 mM^−1^ s^−1^ at 0.47 T and 37 °C, when detecting Zn^2+^ in the presence of HSA (3 times the value obtained for the original probe, **GdP10**) [52].

Our team has also developed a series of probes using derivatives of the DPA motif for Zn^2+^ detection, **GdP16–19** (Figure 6) [53,54,55]. In this case, the Gd^3+^ complexing unit is based on a pyridine motif. It has been shown that the Gd^3+^ bishydrate complex has optimized MRI properties due to its two water molecules in the first coordination sphere, while retaining good thermodynamic and kinetic stability [56]. In addition, ternary complex formation with physiological anions has not been observed [57]. For the compounds **GdP16** and **GdP17**, the relaxivity first increases when 0.5 equivalent of Zn^2+^ is added and then decreases at higher equivalents. The determination of microscopic parameters by independent methods has shown unambiguously that *q* and *k*_ex_ values are not affected by the presence of Zn^2+^, whereas the diffusion coefficient of the species is. This reflects the formation of a dimeric species in the presence of Zn^2+^, which was confirmed by ^1^H NMR spectroscopy. As for previous systems, a good selectivity for Zn^2+^ vs other physiological cations was observed, although a response to Cu^2+^ was detected. As stated above, the non-monotonic response to Zn^2+^ is not optimal for Zn^2+^ quantification, so the Zn^2+^ binding unit was modified in **GdP18** and **GdP19** to minimize the formation of dimeric species [54,55]. With these systems, a response to Zn^2+^ is observed in the presence of HSA, due to different HSA binding affinities of the complexes in the presence or absence of Zn^2+^.

The Zn^2+^ binding affinity is a crucial parameter as it should be high enough to detect signal changes, but not too high to cause depletion of Zn^2+^ in the body. Ideally, the affinity should be in the same range as the local concentration being detected, with interest now on developing systems with different Zn^2+^ affinities in order to explore different Zn^2+^ concentrations. Although the determination methods are different, some general rules can be seen with the systems previously described. When the Zn^2+^ binding unit is based on an iminodiacetate, the affinity for Zn^2+^ is around Log *K*_Zn_ = 3.5–4 (*K*_D_ ≈ 0.1 mM), and when it is a derivative of DPA modified with carboxylate function, Log *K*_Zn_ = 7–8 (*K*_D_ ≈ 10–100 nM) [41]. When the DPA is modified by the addition of a carbon atom (**GdP20**), lower affinities are obtained with Log *K*_Zn_ = 5.6 (*K*_D_ ≈ 2 µM) [58]. Other binding units with different affinities have also been explored, including pyrazoline groups (**GdP21**) with Log *K*_Zn_ = 3 (*K*_D_ ≈ 400 µM), quinoline (**GdP22–24**) with Log *K*_Zn_ = 5–6 (*K*_D_ ≈ µM), or macrocyclic NO2A (**GdP25**) with a very high affinity for Zn^2+^ (Log *K*_Zn_ ≈ 17) [41].

**GdP20** and **GdP21** (Figure 6) function on the same basis as **GdP10** and detect Zn^2+^ in the presence of HSA on the basis of τ_R_ variation. The low binding affinity of **GdP21** for Zn^2+^ is however problematic as a competition with direct Zn^2+^ binding to HSA is present. This does not seem to be observed in the case of **GdP20** although the affinity for Zn^2+^ is in the same range as that of HSA. For **GdP22** (Figure 6) bearing an amidoquinoline group, a low relaxivity of 4.2 mM^−1^ s^−1^ without zinc at 9.4 T suggests that only one water molecule is directly coordinated to Gd^3+^. In the presence of 0.5 equivalent of Zn^2+^, the relaxivity increased up to 6.6 mM^−1^ s^−1^ which represents a 60% increase in relaxivity. Then the relaxivity decreased with the further addition of Zn^2+^. This was explained by the formation of a dimeric species as the hydration state of the corresponding Eu^3+^ complex does not change upon Zn^2+^ addition [59]. The same phenomenon (a 55% relaxivity increase upon Zn^2+^ binding through the formation of a dimeric species) was observed for **GdP23** (Figure 6) where the sulfoquinoline is in the ortho position of the benzene ring [60]. With further Zn^2+^ addition, the formation of the **GdP23Zn** is concomitant with a relaxivity decrease. To circumvent the problem and prevent the formation of the dimeric species, **GdP24** (Figure 6) was successfully designed. In this case, the sulfoquinoline is in the para position of the benzene, far away from the amide, leading to the formation of only **(GdP24)_2_Zn** with an affinity for Zn^2+^ in the pM range [61].

Finally, the detection of Zn^2+^ with **GdP25** (Figure 6) bearing a NO2A (1,4,7-triazacyclononane-1,4-diacetate) moiety, is based on a *q* change [62]. This has been attributed to an increase in the coordination number of Gd^3+^ when binding Zn^2+^, and not to direct coordination of the Zn^2+^ binding unit to Gd^3+^ in the absence of Zn^2+^. The explanation for the increase in the number of water molecules is not obvious since the amide remains coordinated to Gd^3+^ during Zn^2+^ binding, and it was ascribed to the increase in the positive charge of the complex upon Zn^2+^ binding.

It should be mentioned that outside Ln^3+^ probes, a porphyrinic Mn^3+^ contrast agent **MnP26** (Figure 6) has also been developed for Zn^2+^ detection [63]. It is a dual fluorescence and MRI probe, where the fluorescence is switched on upon Zn^2+^ addition, while the relaxivity surprisingly decreases by ca. 25%.

### 4.3. Small Molecular Probes for Cu^2+^ Detection

**Figure 7:**
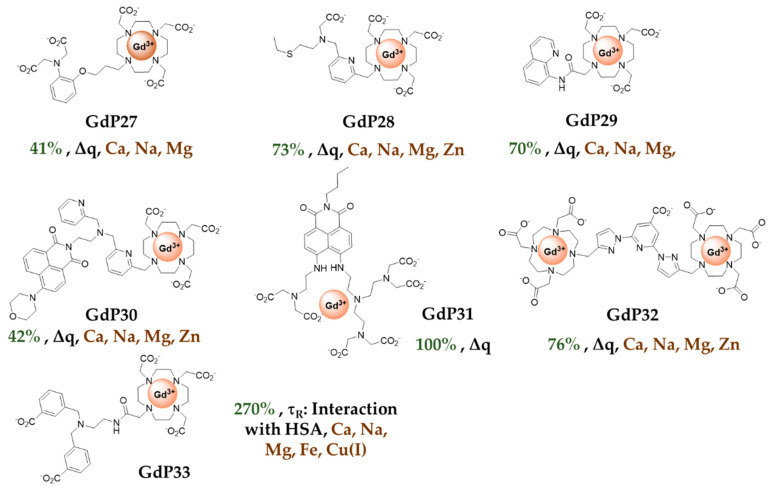
Copper responsive MRI contrast agents: In green (maximum Δr_1_), black (mechanism of response), brown (type of Cu^2+^ response and selectivity).

Some examples of Cu^2+^ detection by MRI have appeared in the literature in the past 10 to 15 years, nevertheless, the research on Cu^2+^-responsive probes is still in its early development compared to the development of Zn^2+^ responsive probes. The first example was proposed by Chang et al., **GdP27** (Figure 7), which is very similar to **GdP4** (Figure 6) [19]. An iminodiacetate moiety is used for Cu^2+^ complexation and a DO3A unit for Gd^3+^ complexation. This probe exhibits a 41% increase in relaxivity upon Cu^2+^ complexation, with a *K_D_* value of 167 µM (affinity for Cu^2+^: Log *K*_Cu_ = 3.8) but has a poor selectivity for Cu^2+^, particularly vs Zn^2+^. To overcome this problem a series of probes containing thiol functions were developed [64]. These softer donor atoms are adapted to the detection of a softer metal ion such as Cu^+^, and indeed most of the probes containing only thiol and amine functions respond only to Cu^+^. **GdP28** (Figure 7), while those containing both thiol and carboxylate functions respond equally to Cu^+^ and Cu^2+^. A 73% increase in relaxivity was obtained upon Cu^2+^ binding due to a change in the Gd^3+^ coordination sphere. A good selectivity vs physiological cations, including Zn^2+^, is obtained with a surprisingly high affinity for Cu^2+^, Log *K*_Cu_ = 15 (*K*_D_ = 0.99 fM). These systems are based on a DO3A unit for Gd^3+^ complexation, and as stated above, competition with physiological anions can be detrimental, which was observed for a similar Cu^+^ responsive complex.

As for Zn^2+^, an amidoquinoline moiety was proposed for Cu^2+^ detection, **GdP29** (Figure 7), and a relaxivity enhancement of ca. 70% was observed linked to an increase in the *q*-value from 1 to 2. This relaxivity increase is partially lost in the presence of physiological anions such as phosphate, carbonate and citrate, due to the replacement of water molecules on the DO3A moiety in the presence of Cu^2+^ [65]. Zn^2+^ selectivity is also not totally convincing as a response is observed in the presence of 10-fold excess of Zn^2+^.

Duan et al. developed a bimodal Cu^2+^-responsive contrast agent, **GdP30** (Figure 7). A GdDO3A complex is linked to a naphthalimide chromophore via a DPA unit [66]. A relaxivity increase of 42% is observed upon Cu^2+^ binding, concomitant with a fluorescence quenching. Surprisingly, this relatively modest relaxivity increase is accompanied by a Δ*q* of more than 2, as measured by luminescence lifetimes of the corresponding Tb^3+^ complex, and attributed to steric hindrance restricting water access to Gd^3+^ in the absence of Cu^2+^. Interestingly a good selectivity vs other physiological cations is observed, including Zn^2+^, which is explained by the presence of the naphthalimide moiety restricting the coordinating ability of DPA for Zn^2+^ and favoring more stable complexes such as those formed with Cu^2+^. An apparent binding constant Log *K*_Cu_ = 4.9 (*K*_D_ = 11.8 µM) was determined by relaxivity measurements. This is lower than the affinity of DPA alone for Cu^2+^ (log *K*_Cu_ = 9.31) [67], which is consistent with the presence of the naphthalimide moiety.

Another bimodal probe containing a naphthalimide moiety, **GdP31** (Figure 7), has been reported [68]. A low relaxivity of 2.0 mM^−1^ s^−1^ (60 MHz, 25 °C) is observed in the absence of Cu^2+^, consistent with the absence of a water molecule directly coordinated to Gd^3+^. Upon Cu^2+^ coordination, 100% relaxivity increase is observed. However, in vitro, this increase is observed when up to 10 eq of Cu^2+^ is added, and at such relaxivities, the paramagnetism of Cu^2+^ might not be negligible and should be taken into account.

Xu et al. developed a dinuclear Gd^3+^ probe, **GdP32** (Figure 7) [69]. A relaxivity enhancement of ca. 75% upon Cu^2+^ binding is observed with good selectivity for Cu^2+^ vs other physiological cations including Zn^2+^. The mechanism of response is based on a *q*-change upon Cu^2+^ binding but the response is partially lost in the presence of physiological anions such as phosphate.

More recently, Sherry et al., proposed a new Gd^3+^-based copper-responsive MRI contrast agent **GdP33** (Figure 7) [70]. Upon the addition of one equivalent of Cu^2+^, the probe exhibited an increase of 43% in relaxivity at 20 MHz, and the affinity was determined by fluorescence as Log *K*_Cu_ = 4.08 (*K*_D_ = 84 µM). The relaxivity increase is amplified to 270% in the presence of HSA. Cu^2+^ is bound to the N-terminal site of HSA [71] with a log *K*_Cu_ = 13 [72], suggesting that Cu^2+^ remains in this domain of the protein in the presence of **GdP33**. This is confirmed by XANES experiments, showing very similar signatures of Cu-HSA and **GdP33-Cu-HSA**, supporting the fact that Cu^2+^ remains in the same environment whether **GdP33** is present or not. Therefore, this very high relaxivity increase is surprising, as the formation of the ternary complex seems relatively weak, but is explained by the slow tumbling dynamics of the system. Moreover, the complex responds to Zn^2+^ to a lesser extent but this can be problematic due to the high Zn^2+^ concentrations in vivo. The Cu^2+^ response is also altered in the presence of PBS.

### 4.4. Selectivity Issues for Zn^2+^ and Cu^2+^ Detection

The development of Cu^2+^-responsive probes is much less explored than that of Zn^2+^ sensitive probes. First*,* important relaxivity responses must be achieved as Cu^2+^ will be present typically in lower concentration*s* compared to the contrast agent, so the response will be “diluted” with the presence of Cu-free contrast agent. Another important point for the practical use of such probes is to obtain a good chemical selectivity vs Zn^2+^. Indeed, the Cu-free contrast agent will certainly bind to Zn^2+^ if the affinity is not negligible, which will hamper the Cu^2+^ response if Zn^2+^ systems are also responsive. The chemical selectivity however is not easy to achieve. This is illustrated in Table 1, which shows that the binding sites explored for Zn^2+^ and Cu^2+^ detection are often based on similar chemical structures. Responses to Zn^2+^ are rarely selective vs Cu^2+^, although it should be noted that for **GdP9** a better selectivity is obtained due *to* the addition of extra coordinating atoms [49]. This is not problematic for practical Zn^2+^ detection*,* as Zn^2+^ is much more concentrated in vivo compared to Cu^2+^. However, this is not true for Cu^2+^ detection. Only three Cu^2+^ contrast agents are selective vs Zn^2+^*:* **GdP30**, **GdP28**, and **GdP32** (see Table 1). **GdP30***,* based on a DPA unit*,* exhibits a surprising selectivity towards Cu^2+^. However, the authors explained that the presence of a Naphthalimide moiety directly linked to the DPA, decreases the affinity of the Zn^2+^ ion vs strongly coordinating Cu^2+^ ions, making the “switch” of one pyridine unit for Gd^3+^ to Cu^2+^ favored compared to Zn^2+^. Decreasing the affinity for both ions is certainly a route to explore as binding to Cu^2+^ is typically stronger, however, it is not easy to predict and practically achieve. **GdP*28***, with the presence of softer atoms such as thiols*,* is selective for Cu^2+^ vs Zn^2+^ however, it also responds to softer Cu^+^. Finally, the design of **GdP32** seems interesting to obtain a good selectivity for Cu^2+^ vs Zn^2+^. The responses of **GdP32**, **GdP28** and **GdP30** is based on a change of the hydration sphere of Gd^3+^ in the presence of Cu^2+^. The Gd^3+^ coordination sphere is based on a DO3A unit, which is sensitive to anion binding. Therefore, in practice, the relaxivity enhancement observed will be partially annihilated by the binding of anions such as citrate, phosphate, etc. Moreover, the stability of the probes towards cation transmetallation would certainly be a crucial point to assess for their practical in vivo use.

### 4.5. Bio-Inspired Probes

The use of a bioinspired system represents an alternative route to the development of cation responsive contrast agents. So far, there is only one example of such a system for Zn^2+^ detection.

Instead of appending a small Zn^2+^-binding moiety to a Gd^3+^ chelate, the idea is to use a natural zinc-binding scaffold, such as a zinc finger peptide, to which a stable Gd^3+^ chelate has been attached. Classical zinc-finger peptides are short peptides (25–30 amino acids) sharing a common sequence of the form (Tyr/Phe)-Xaa-Cys-Xaa_2/4_-Cys-Xaa_3_-Phe-Xaa_5_-Leu-Xaa_2_-His-Xaa_3_-His, where Xaa can be any amino acid [74]. Zinc-binding occurs through the two Cys and two His residues of the sequence. In the absence of Zn^2+^, the peptides have no defined conformation, while a folding occurs when bound to Zn^2+^ and the peptide adopts a compact βαα conformation (two β-sheets followed by an α-helix).

**Figure 8:**
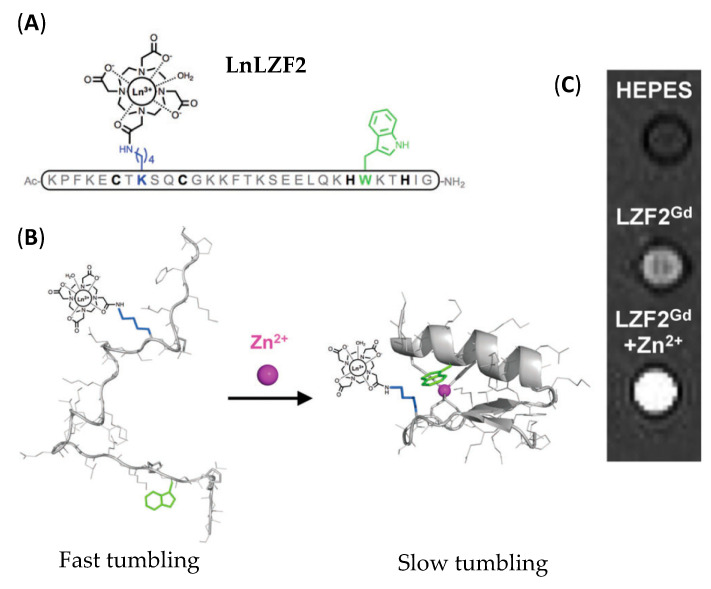
Bioinspired zinc finger peptide and principle for zinc detection by Magnetic resonance imaging (MRI): (**A**) Structure of bioinspired zinc-finger peptide **LnLZF2**; (**B**) Representation of the folding of **LnLZF2** upon Zn^2+^ binding; (**C**) T_1_-weighted MR images of phantoms containing Zn-free ad Zn-loaded **GdLZF2** at 1.5 T. Adapted with permission from Ref [75]; published by The Royal Society of Chemistry, 2018.

A 30-amino acid peptide based on the zinc-finger scaffold and to which a DOTAMA (DOTA monoamide) ligand for Ln^3+^ complexation was added, as well as tryptophan to sensitize Ln^3+^ luminescence (**LnLZF2**, Figure 8) was recently developped [75]. This probe is therefore a dual-mode system as the Zn^2+^ can be detected by luminescence or MRI depending on the chosen Ln^3+^, Tb^3+^ and Gd^3+^, respectively. The detection is based on the change of conformation of the peptide upon folding. Indeed, for luminescence, Tb^3+^ needs to be sensitized through the antenna effect by Trp. In the absence of Zn^2+^, Trp and TbDOTAMA are too far away to observe any energy transfer, whereas upon binding, the two entities are brought in close proximity and the energy transfer can occur, leading to a switch on the luminescent signal. Concerning the Gd^3+^ complex, in the absence of Zn^2+^, the unstructured peptide is flexible, whereas, upon binding, the system becomes more compact, leading to an increase in rotational correlation time τ_R_, and thus an increased relaxivity. A relaxivity increase of 40% is obtained at 37 °C and 20 MHz. A temperature-dependent study indicates that the system undergoes a change in dynamics upon Zn^2+^ binding (rotation is the factor limiting relaxivity in the absence of Zn^2+^, whereas in the presence of Zn^2+^, the slow water exchange rate becomes limiting). Nuclear Magnetic Relaxation Dispersion (NMRD) measurements, combined with ^17^O NMR spectroscopy unambiguously show the system is more rigid in the presence of Zn^2+^, as was foreseen. Phantom images could be registered at 1.5 T and 9.4 T. At 1.5 T a brighter image is obtained in the presence of Zn^2+^ (Figure 8) whereas, at 9.4 T, the brighter image is observed in the absence of Zn^2+^, which is consistent with the NMRD profiles. The system is highly selective for Zn^2+^ vs other cations such as Mg^2+^, Cu^2+^, Ca^2+^, Mn^2+^ and Fe^2+^, and the dissociation constant is in the picomolar range (2.5 × 10^−12^ pM) as determined by luminescence.

## 5. Other Responsive Contrast Agents for Zn^2+^ and Cu^2+^ Detection

### 5.1. ParaCEST Probes

**Figure 9:**
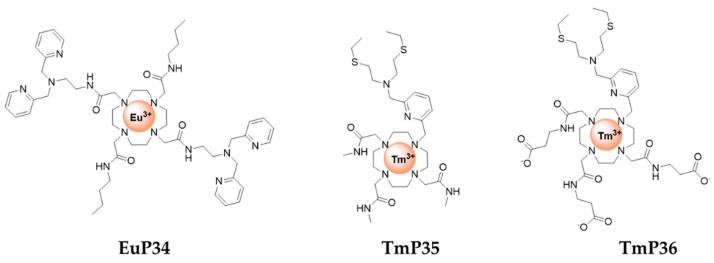
ParaCEST MRI contrast agents for zinc detection.

Some ParaCEST probes have also been developed for Zn^2+^ detection. The first example, **EuP34** (Figure 9), is a DOTA tetramide ligand [76]. In the absence of Zn^2+^, the water molecule coordinated to Eu^3+^ gives a CEST effect at 50 ppm. In the presence of Zn^2+^ at pH 7, this effect is modified (decreased), and at pH 8 the CEST effect disappears. This may be explained by the formation of a Zn-hydroxo complex, which catalyses the exchange of the water molecule (or proton) coordinated to Eu^3+^. The system shows good selectivity for Zn^2+^ over Ca^2+^ and Mg^2+^.

More recently, **TmP35** and **TmP36** (Figure 9) were proposed as ON/OFF responsive ParaCEST MRI contrast agents for zinc and copper detection [77]. The ParaCEST effect originates from the amide proton and is switched OFF upon Zn^2+^, Cu^+^ or Cu^2+^ binding. The formation of TmP/Zn 2/1 complex, TmP/Cu 2/1 complex for Cu^2+^ and 1/1 complex for Cu^+^ is responsible for the faster exchange of amide protons and disappearance of the CEST signal. Obviously, the selectivity of the system is poor and the switch OFF CEST signal remains a difficulty for any practical application.

The rare development of ParaCEST probes for Zn^2+^ and Cu^2+^ detection can be explained by the difficulty to rationalize CEST changes upon cation binding and to design successful systems. Moreover, the low concentration of “free” metal ion combined with the low sensitivity of ParaCEST can be problematic for practical in vivo detection. Finally, in the case of Cu^2+^, the paramagnetism is certainly an additional difficulty for such detection.

### 5.2. Parashift Probes

**Figure 10:**
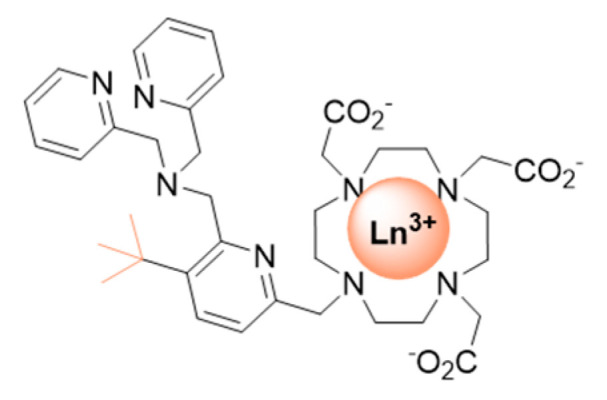
Parashift probe for Zn^2+^ detection.

Very recently in a proof of concept study, **LnP37** (Figure 10) was proposed to signal changes in Zn^2+^ concentrations by chemical shift modification and luminescent emission changes [78]. The *tert*-butyl group serves as the parashift reporter. The complex has no coordinated water molecule in the absence of Zn^2+^ (as demonstrated by luminescence lifetime measurements of the Eu^3+^ and Tb^3+^ complexes) and becomes monohydrated upon Zn^2+^ coordination. The emission spectra of **EuP37** is greatly affected upon Zn^2+^ coordination, confirming important modification in the Eu^3+^ coordination sphere. **TbP37**, **DyP37**, and **TmP37** have paramagnetically shifted *tert*-butyl proton resonances of −30.2 ppm, −36.5 ppm, and +17 ppm, respectively. The addition of Zn^2+^ resulted in the appearance of new *tert*-butyl peaks. The relaxation rates of those are greatly decreased, which is probably the result of an increased distance between the *tert*-butyl protons and the paramagnetic center.

#### ^19^F MRI Probes

**Figure 11:**
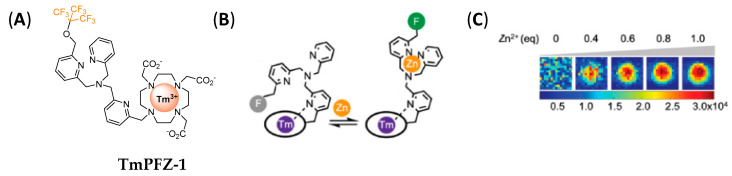
(**A**) Chemical structure of **TmPFZ-1**; (**B**) Schematic representation of the conformational exchange within **TmPFZ-1** upon Zn^2+^ complexation; (**C**) ^19^F MRI images of **TmPFZ-1** (4 mM) in the presence of different Zn^2+^ equivalents. Adapted with permission from Ref [81]; published by The Royal Society of Chemistry, 2020.

In the past few years, there has been renewed interest in heteronuclear MRI, particularly ^19^F MRI. ^19^F is the most promising imaging nucleus due to the natural abundance of ^19^F with spin 1/2, and the negligible presence of endogenous signals, since only a trace amount of ^19^F are present in the body. However, low doses of fluorinated agents and the low efficiency of T_1_ lead to long acquisition times, and the sensitivity of ^19^F MRI may be a problem. The use of dual-modality probes and large molecules with high fluorine content have been explored to mitigate these issues. Many ^19^F probes have been developed, including organic molecules, metal complexes, or nanoparticles [79]. Responsive paramagnetic complexes have also flourished, particularly based on transition metal ions for redox or pH sensing [80]. Recently, a Tm^3+^ complex, **TmPFZ-1** bearing nine fluorine atoms on the DPA moiety was developed by Que et al. (Figure 11) [81]. In the absence of Zn^2+^, the system is highly flexible and the probe is not detectable by ^19^F MRI. Upon Zn^2+^ coordination, a rigidification of the system is observed and one major, accompanied by three minor, ^19^F NMR signals appear. These correspond to different diastereoisomers of the probe. Zn^2+^ could be detected with ^19^F MRI, with a detection limit of 180 µM in vitro.

## 6. In Vivo Detection of Zn^2+^ and Cu^2+^

Despite numerous examples of Zn^2+^, and to a lesser extent Cu^2+^ responsive probes, the in vivo applications of such probes are only at the early stages of development.

For example, **MnP26** (Figure 6) is cell-permeable and cellular studies have shown that cells treated with Zn^2+^ show a greater contrast than those without Zn^2+^, contrary to what was observed in vitro (relaxivity decrease) [63]. The in vivo behavior of the compound was further investigated by intracranial injection in living rats. A correlation between contrast enhancement and Zn^2+^-rich area of the brain was found [82].

**Figure 12:**
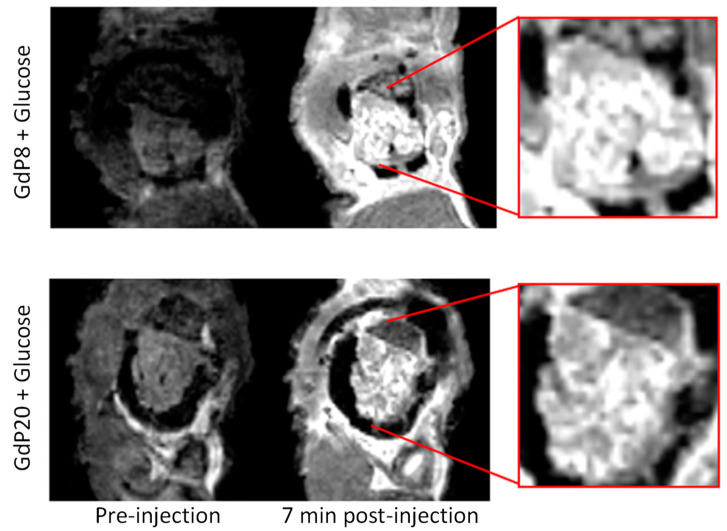
Three-dimensional T_1_-weighted MR images of mouse pancreas pre or post-injection of **GdP8** or **GdP20** plus glucose (for Zn^2+^ release). Reproduced with permission from Ref [58]; published by American Chemical Society, 2018.

Given the high concentration of Zn^2+^ in the pancreas, and the easier access (no crossing of the blood–brain barrier is necessary), this organ has also been imaged with Zn^2+^ responsive probes. This was the case with **GdP22** (Figure 6), which was demonstrated to be cell-permeable, and non-toxic (on MIN6 and HEK 293 cells). In vivo MR images were acquired on healthy C57/BL6 mice after injection of 0.1 mmol kg^−1^ of **GdP22** and show uptake of the agent in the pancreas, likely reflecting the accumulation of the agent where free Zn^2+^ is present [59]. The most important in vivo work on Zn^2+^ detection was certainly performed by Sherry and coworkers using **GdP10** (Figure 6). First, this probe has been used in vivo on mice to image β-cells from the pancreas. Conversely to the previous example, in this case, a glucose stimulation was used to release Zn^2+^ from the β-cells together with insulin. They showed that a greater contrast was observed: (1) upon glucose stimulation compared to saline injection; (2) after glucose stimulation on a 24-week old mouse with twelve weeks of a high-fat diet compared to a low-fat diet; (3) after glucose stimulation on normal mice rather than STZ treated mice (model of type I diabetes), where STZ (streptozotocin) is a toxin for β-cells [83]. As **GdP12** was found to be optimized in vitro compared to **GdP10**, images of mice pancreas after glucose stimulation were compared in the presence of **GdP10** and **GdP12**. However, no significant differences in contrast enhancement had been observed. This could be explained by the difficulty in the selection of the ROI (region of interest) of the pancreas given the fact that it is not a solid organ, and/or by the field of detection, which is 0.5 T in vitro and 9.4 T in vivo [52]. Recently, it was demonstrated that the use of lower-affinity zinc sensors, such as **GdP20** (Figure 6), improved the imaging of Zn^2+^ from mouse pancreas [58]. Indeed, they compared images obtained after glucose stimulation from mouse pancreas with **GdP20** and **GdP8**, using an MR-compatible window to hold the pancreas in place. The two agents have an affinity for Zn^2+^ of Log *K*_Zn_ = 5.6 and 6.9, respectively, while the affinity of Zn^2+^ for HSA is Log *K*_Zn_ = 7.5. Upon glucose stimulation, a non-uniform signal enhancement was shown across the pancreas with “hot spots” (which could correspond to first-responder islets) which were evidenced more easily using **GdP20** (Figure 12). This was attributed to an important “background signal” using **GdP8**. The change in intensity upon injection of the contrast agent is nearly two times more important with **GdP8** compared to **GdP20** without glucose stimulation. Model binding predictions confirmed these experimental findings.

The prostate also contains a very high amount of Zn^2+^, which has been known to be an important biomarker to differentiate between healthy tissue, benign prostatic hyperplasia and prostate cancer [84]. Non-invasive imaging of Zn^2+^ using **GdP11** (Figure 6) has shown the possibility of identifying small prostatic malignant lesions at a very early stage of 11 weeks, where it might be difficult to detect cancer by classical MRI methods [85]. It was demonstrated that the secretion of Zn^2+^ from prostate tissue is stimulated by glucose in fasting mice and that this release can be monitored by MRI. The lower content of Zn^2+^ in transgenic adenocarcinoma of the mouse prostate model was clearly shown. More recently, the Zn^2+^ release was monitored across the prostate during the development of the malignancy using the zinc-sensitive contrast agent **GdP20** by synchrotron radiation X-ray fluorescence [86]. Quantitative measurements show that the lateral lobe of the mouse prostate is crucial as it accumulates the high Zn^2+^ content and the loss of Zn^2+^ during the development of the tumor is also observed in this area. Colocalisation of Zn^2+^ and Gd^3+^ also confirmed that glucose initiates the secretion of Zn^2+^ from intracellular compartments of the prostate to the extracellular media where it binds to the responsive contrast agent. Interestingly, they also observed that Gd^3+^ content is not the same in the prostate whether the mouse is healthy or not and has been injected with glucose.

As Cu^2+^ is less concentrated than Zn^2+^, the in vivo detection of Cu^2+^ is even more challenging and very few probes have attained in vivo studies. **GdP29** was used in Zebrafish, however, it was the fluorescence properties of the system (quenching of fluorescence in the presence of Cu^2+^) that were exploited [66].

Cu^2+^ was detected using **GdP33** by MRI in vivo in mice. Of the contrast enhancement, 25% was observed in the liver of the mice upon injection of the contrast agent, while a lower contrast enhancement was observed for mice treated with a Cu^2+^ chelator [70]. The Gd^3+^ and Cu^2+^ content was then determined by ICP analysis. An interesting addition to this study would be to measure the Zn^2+^ content to ascertain if it was altered, knowing that the contrast agent also responds to Zn^2+^.

Finally, the in vivo applications of ParaCEST or parashift contrast agents are only beginning to be researched compared to T_1_-contrast agents. However, the feasibility of the in vivo detection of such responsive probes has been proven, especially for pH detection [38,87]. Concerning cation detection in general, no responsive probes have been applied in vivo. This could be explained by the fact that very few probes have been developed for this purpose so far and optimization is needed before in vivo translation.

## 7. Fast-Field Cycling MRI: A Way to Improve Cation Detection at High Fields

**Figure 13:**
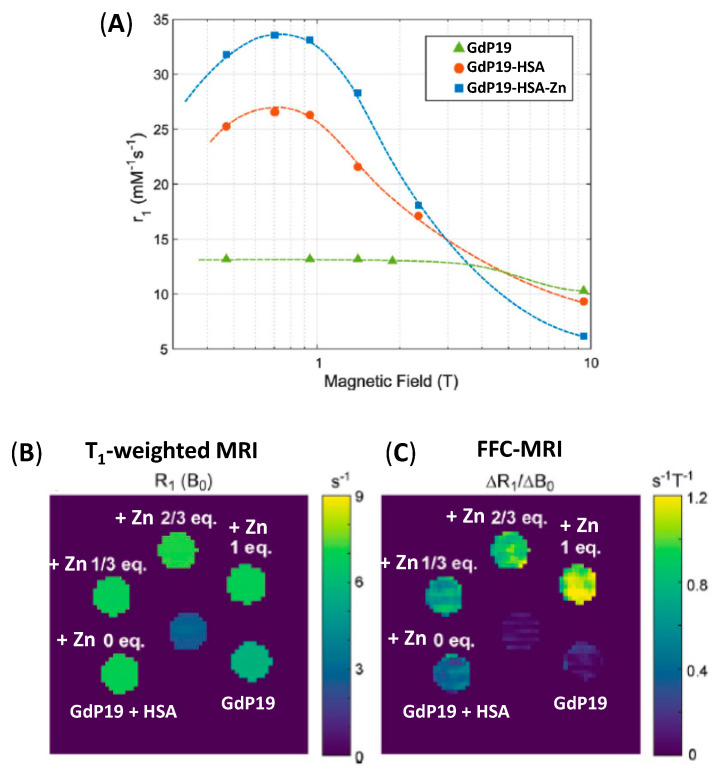
(**A**) ^1^H NMRD profile of **GdP19** alone, in the presence of human serum albumin (HSA), and HAS + Zn^2+^; (**B**) R_1_ map obtained at 2.89 T, the nominal field strength of the MRI system; (**C**) ΔR_1_/ΔB_0_ map obtained from subtracting R_1_ maps at 2.99 T and 2.79 T. Adapted with permission from Ref [55]; published by Wiley-VCH, 2018.

Classically, the T_1_-based contrast agents described above utilise T_1_-weighted images at a given field. When the mechanism for cation (or any biomarker) detection is based on a change in the hydration number *q*, the relaxivity changes remain relatively independent of the field. However, when the mechanism is based on a change in the rotational correlation time τ_R_, the relaxivity response is more important at the medium field (20–80 MHz; 0.5–2 T), and diminishes or even vanishes at higher fields. It is often interesting to work at high fields where the resolution is better but at these fields, the effect of long τ_R_ is greatly diminished. For example, most of the studies performed with **GdP10** (Figure 6) for Zn^2+^ detection on the pancreas and the prostate were done at 9.4 T [52,83,85], while the Cu^2+^ detection study in the presence of **GdP33** was done at 4.7 T [70]. These contrast agents detect Zn^2+^ or Cu^2+^ in the presence of HSA, through the formation of a ternary complex. The in vitro studies were generally performed around 20 MHz (0.5 T) and for example, for **GdP20** the relaxivity increase upon Zn^2+^ binding is more than 100% at 0.5 T, while it is less than 8% at 9.4 T, the imaging field. The situation is even worst for **GdP8** for which the response drops from 200% at 0.5 T to 8% at 9.4 T [58]. In general, to understand in vivo results, it is important to perform in vitro studies at the same field. Moreover, it is clear that the development of high-performance contrast agents with the best response upon cation binding can be biased and/or annihilated depending on the detection field. Therefore, the development of new methods for high-field detection of biomarkers for which the response is based on a change in the rotational correlation time τ_R_ is needed. Fast field-cycling magnetic resonance imaging (FFC-MRI) is a novel strategy in MRI that takes specific advantage of the magnetic field dependency of relaxivity, rather than on the relaxivity value at a given field. In FFC-MRI the magnetic field is changed during the imaging sequence, while in conventional MRI the main magnetic field is fixed [88]. This method is particularly powerful to visualize macromolecules with long τ_R_ and high R_1_ (=1/T_1_, relaxation rate) dispersion as the information contained in the images is based on the partial derivative of the longitudinal relaxation rate with respect to the magnetic field, ΔR_1_/ΔB_0_. It was successfully used to image probes that exhibit a strong relaxivity response upon protein binding, especially in the case of HSA [89]. We recently demonstrated the potential of such a technique for Zn^2+^ detection using **GdP19** [55]. It is clear from Figure 13B that at 3 T, no difference in the sample is seen whether Zn^2+^ is present or not. This is in accordance with the NMRD profile, where no relaxivity difference is observed in this field (Figure 13A). However, at 3 T, the derivative of R_1_ as a function of the magnetic field (which is represented by the slope of the NMRD profile at this field) is very different. This is reflected by the different contrast observed in the absence or in the presence of various amounts of Zn^2+^ using FFC-MRI (Figure 13C).

This is very general and can be applied to the detection of any biomarker based on a change of the rotational correlation time. This also illustrates that depending on the detection technique used, biomarkers can be switched ON or OFF and sensitivity can be increased.

## 8. Quantification Methods for Cation Detection

Another important issue to solve is the quantitative detection of the cation. Indeed, the early detection of diseases can be performed via the alteration of cation distribution, but in order to follow this distribution, the best method would be to quantitatively assess their concentration repeatedly over time.

MRI is not a quantitative technique in the sense that the signal observed depends both on the presence of the cation (or biomarker) being detected, but also on the local concentration of the agent, which is not known in vivo. This can be problematic as a difference of contrast can be attributed to a different concentration of cation and/or the distribution of the contrast agent. For example, the detection of Zn^2+^ in the pancreas has been performed comparing mice with glucose stimulation or without [83]. Several difficulties can occur: (1) selecting the same ROI because the pancreas is a very diffuse organ; (2) variations can occur between different mice; (3) the distribution of the agent can be different in the presence or absence of glucose. The first problem has been minimized by using chambers to select the same slice of the pancreas [58]. The variations within the mouse can also be taken into account by imaging a sufficient number of animals (at least 5), but the differences of distribution of the agent are impossible to detect if another method for local quantification of the agent is not performed. The same problems can occur for the detection of Zn^2+^ for early prostate tumor diagnosis. There is no proof that the distribution of the agent will be the same in healthy versus malignant tissues. Indeed quantitative methods using synchrotron X-Ray fluorescence (a destructive technique) have recently shown great variability in the Gd^3+^ concentration from the contrast agent between healthy and malignant prostate tissue [16].

The example of MnP26 is also appealing in the sense that the results observed in the buffer and in cells are opposite, suggesting that the cellular uptake of the Zn^2+^-bound and Zn^2+^-free form of the complexes is different [82].

These examples highlight the importance of developing robust quantification methods in vivo for cation detection. If quantification methods have been proposed so far, they have been essentially devoted to pH detection, and cannot be always translated to cation detection. Because the problem of quantification is the same for all metal ions and very few systems have been proposed so far, the examples given below will not only focus on Zn^2+^ or Cu^2+^ to illustrate the variety of possibilities.

Concerning pH quantification so far, a R_2_/R_1_ ratiometric method was proposed, where R_2_ and R_1_ are the transverse and longitudinal relaxation rates of the water protons [90]. Given the relatively large inherent R_2_ in living tissues relative to R_1_, the in vivo feasibility of this method has yet to be proven. To estimate the in vivo concentration of a pH-responsive probe used to map pH values in renal acidosis and brain tumor models, a complex which displays a pH-independent relaxivity behaviour was first injected, assuming identical pharmacokinetics for the two complexes (responsive or not) [91]. This is a strong limitation to the method as even small structural changes can lead to dramatic differences in biodistribution [92]. The use of bimodal probes was also explored to combine MRI with a quantitative technique such as SPECT or PET. A ^18^F PET reporter was introduced in a Gd^3+^-pH-sensitive contrast agent and to overcome sensitivity problems the ^18^F atoms were diluted in ^19^F [93]. The similar properties of Ln^3+^ ions were also exploited for pH quantification. ^166^Ho, active in SPECT, was used as a surrogate of Gd^3+^ and a cocktail of the two complexes was used for in vitro validation of the technique [94]. However, ^166^Ho is produced from ^165^Ho and it is not possible to separate each isotope. This is not problematic for pH detection, but for cations present at µM concentrations, the high quantity of ^165^Ho will annihilate or at least strongly limit the MRI response.

To circumvent this problem, we recently proposed the first in vitro proof of concept study of cation detection using ^165^Er [54]. ^165^Er is readily obtained from a cyclotron and can be purified from the parent compound ^165^Ho, which is indispensable for reliable quantification. A cocktail of **GdP18** and ^1**65**^**ErP18**, respecting the sensitivity of each technique, was used in vitro in the presence of HSA. Unknown quantities of Zn^2+^ were added to the samples. The activity of each sample was measured using γ-spectrometry and the samples were imaged with a γ-camera. This allows for the determination of ^165^Er^3+^ concentration, and consequently Gd^3+^ concentration by knowing the ^165^Er/Gd ratio. Then, the samples were imaged at 3 T, and using a calibration curve at the same field and temperature, it was possible to determine the exact Zn^2+^ content. The values found with this method were compared to ICP results and values within less than 15% were found, within the experimental error. These encouraging results have now to be proven in vivo.

Our approach was supported recently by an Ln surrogate ^86^Y, a positron emitter, which was used to quantify a whole-body distribution of Gd^3+^ contrast agent [95].

Quantification issues for “frequency-encoded” techniques are less critical. For example, the use of Parashift probes is very convenient for concentration determination. Using **LnP37** (Figure 10), the presence of Zn^2+^ is detected by a change in the chemical shift of the probe resonance. This chemical shift is independent of the concentration of the probe but the ratio of the integral of the Zn^2+^-free and Zn^2+^-bound species is dependent upon Zn^2+^ concentration [78].

^19^F MRI was also used recently as a complementary technique to ^1^H MRI for probe quantification. A perfluoro-15-crown-5-ether was included in the hydrophobic core of a micelle composed of surfactant and an amphiphilic Gd^3+^ complex responsive to Ca^2+^ [96]. The system shows a 30% increase in r_1_ and a 343% increase in r_2_ upon Ca^2+^ binding at 7 T and 25 °C. T_1_-, T_2_- and T_2_/T_1_-weighted images were performed first on phantom samples containing different Gd^3+^ concentrations and diamagnetic samples of trifluoroacetic acid (TFA) for calibration of the ^19^F signal. These studies show that ^19^F MRI images are not affected by the presence of Ca^2+^ so that the ^19^F signal could indeed be used as a concentration reporter. These studies were then performed in vivo with an intracranial injection of the micelle containing the contrast agent in the somatosensory cortex of rats, a vial of TFA serving as a reference. Good signal enhancements are observed, which enabled the calculation of local Gd^3+^ concentrations. It would have been interesting to use the fluorinated micelle itself for the external calibration (instead of TFA), as the presence of the paramagnetic ion might affect the calibration. Importantly, the fate of the micelle is not entirely known in vivo.

**Figure 14:**
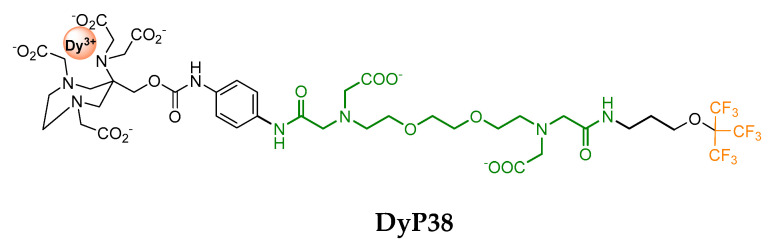
Paramagnetic probe sensitive to Ca^2+^ and used as a cocktail with the corresponding YP38 for Ca^2+^ quantification.

The same group developed a ^19^F Ca^2+^ responsive probe **DyP38** (Figure 14) which can be used for quantification [97]. The Ca^2+^ responsive unit was labelled with nine equivalent fluorine atoms and a DyAAZTA complex. The probe was designed to display a single ^19^F signal in the absence of Ca^2+^ due to the presence of a single isomer of the Ln^3+^ complex. Upon Ca^2+^ coordination, the ^19^F atoms come closer to the paramagnetic Dy^3+^ leading to a shortened T_1_ and a shift of the resonance. The use of a diamagnetic Y^3+^ analog, insensitive to Ca^2+^, enabled probe quantification, and as a consequence Ca^2+^ quantification.

Finally, the ^1^H parashift probe **LnP37** (Figure 10) was used to detect the change in Zn^2+^ concentration. Indeed, the presence of Zn^2+^ is detected by a change in the chemical shift of the probe resonance. This chemical shift is independent of the concentration of the probe but the ratio of the integral of the Zn^2+^-free and Zn^2+^-bound species is dependent upon Zn^2+^ concentration [78].

## 9. Conclusions

Recent years have witnessed tremendous efforts in the development and in vitro characterization of metal-based MRI responsive probes for Zn^2+^ and Cu^2+^ detection. The first successful translation of Zn^2+^ responsive probes in vivo encouraged original chemical design to meet challenges in this field such as: (1) optimal affinity for the cation to sense; and (2) quantification of the probe. Another key challenge is the selectivity, which is particularly important due to the chemical similarity between Zn^2+^ and Cu^2+^. The development of bio-inspired probes can be an interesting alternative. Nevertheless, this selectivity problem coupled with the low in vivo Cu^2+^ concentrations is certainly preventing further practical Cu^2+^ detection in vivo.

The vast majority of the probes developed so far are Gd^3+^-based contrast agents for traditional relaxation-based ^1^H detection. In this case, the chemical selectivity of the sensing unit is crucial to achieving accurate detection. The development of new binding units with good selectivity for Cu^2+^ vs Zn^2+^ will be needed. The tight control of the coordination properties of Gd^3+^ and Zn^2+^ or Cu^2+^ to develop systems that respond only to one cation can also be explored, but is not easily predictable. Those systems work on the basis either of a *q*-change or a τ_R_ change mainly through interaction with HSA. In the first case, the possible response to physiological anions in the presence of the cation is an important point to take into account, and will certainly lead to the development of other Gd^3+^ units less sensitive to anions. In the case of HSA interactions, the response will be guided by the difference of affinity of the contrast agent for HSA with and without the cation to sense. In order to optimize those systems, a better understanding of the relationships between the structure of the contrast agent and the interaction with HSA will be required. Deciphering the rationale behind the HSA binding sites which depends on the chemical structure, affinity for Zn^2+^ or Cu^2+^, etc., will also call for future developments.

In order to avoid the toxicity of exogenous Gd^3+^, other bioresponsive probes, such as those developed for pH sensing or redox detection, are currently based on more biocompatible transition metal ions. However, at the concentration needed for MRI exams, these ions would be toxic and also need to be encapsulated in a stable chelate. In the case of cation detection, the question of the kinetic inertness and thermodynamic stability is even more significant due to the possible competition between the MRI active cation and the cation to sense, which is particularly true for Zn^2+^ detection using Mn^2+^ complexes.

^19^F MRI is also appearing in the field of cation detection using paramagnetic complexes both as a direct method for detection exploiting the advantage of the absence of endogenous signal or as a complementary technique for probe quantification.

Finally, alternative mechanisms to traditional relaxation-based ^1^H detection such as Parashift, ParaCEST techniques are starting to be explored. Their frequency-encoded signals rendered the quantification problems probably easier to solve. Future development of such probes will rely on the fine-tuning of the physical–chemical parameters of the probe and a better understanding of the important parameters for successful in vivo translation.

## Figures and Tables

**Table 1:** Partial binding site, mode of detection, affinity and selectivity of T_1_-based probes for Zn^2+^ and Cu^2+^ detection.

Partial Cation Binding Site	Name	Detection of Zn/Cu	*K*_d_ for the Sensed Cation	Δr_1_ (%)	Mode of Detection	Selectivity Zn vs Cu	Selectivity vs Other Cations	Ref.
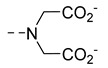	**GdP4**	Zn	240 µM	121	Direct: Δ*q*	No	Ca, Mg	[45]
	**GdP9**	Zn	126 (316 ^a^) µM	70	Direct: Δ*q*	Partially	Ca, Mg, Na, K	[49]
	**GdP27**	Cu	167 µM	41	Direct: Δ*q*	No	Ca, Mg, Na, K	[19]
	**GdP31**	Cu	11 µM	100	Direct: Δ*q*	No	nd	[68]
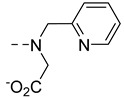	**GdP2**	Zn	nd	−30	Direct: Δ*q*	No	Ca, Mg	[44]
	**GdP16**	Zn	10 nM	20	Direct: τ_R_	No	Ca, Mg	[53]
	**GdP17**	Zn	10 nM	20	Direct: τ_R_	No	Ca, Mg	[53]
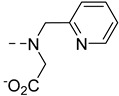	**GdP1**	Zn	nd	−33	Direct: Δ*q*	No	Ca, Mg	[43]
	**GdP8**	Zn	118 nM	200	Interaction with HSA	No	Ca, Mg	[58]
	**GdP10**	Zn	33.6 nM	165	Interaction with HSA	No	Ca, Mg	[47]
	**GdP19**	Zn	nd	25	Interaction with HSA	No	Ca, Mg	[55]
	**MnP26**	Zn	12 nM	−25	nd	No	Ca, Mg	[63]
	**GdP30**	Cu	12 µM	42	Direct: Δ*q*	Yes	Ca, Mg, Na	[66]
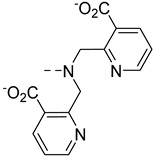	**GdP18**	Zn	126 nM	25	Interaction with HSA	No	Ca, Mg	[53]
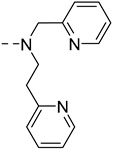	**GdP20**	Zn	2350 nM	100	Interaction with HSA	nd	nd	[58]
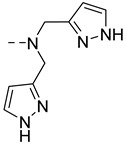	**GdP21**	Zn	379 µM	80	Interaction with HSA	No	Ca, Mg	[73]
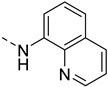	**GdP22**	Zn	22 µM	57	Direct: τ_R_	No	Ca	[59]
	**GdP23**	Zn	476 nM; 2.4 pM ^b^	55	Direct: τ_R_	No	Ca, Mg, Fe	[60]
	**GdP24**	Zn	435 fM ^b^	44	Direct: τ_R_	No	Ca, Mg, Fe	[61]
	**GdP29**	Cu	160 pM	66	Direct: Δ*q*	No	Ca, Mg, Na	[65]
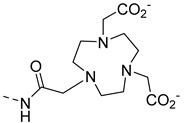	**GdP25**	Zn	nd	150	Direct: Δ*q*	No	Ca, Mg	[62]
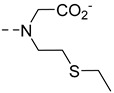	**GdP28**	Cu	0.99 fM	73	Direct: Δ*q*	Yes	Ca, Mg, Na	[64]
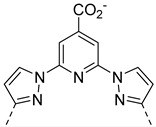	**GdP32**	Cu	nd	76	Direct: Δ*q*	Yes	Ca, Mg, Na	[69]
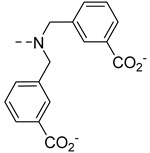	**GdP33**	Cu	84 µM	270	Interaction with HSA	No	Ca, Mg, Na, Fe, Cu(+I)	[70]

^a^ In mouse serum; ^b^ For the formation of (GdP)_2_Zn.
